# Association between mechanical power during one-lung ventilation and pulmonary complications after thoracoscopic lung resection surgery: a prospective observational study

**DOI:** 10.1186/s12871-024-02562-1

**Published:** 2024-05-17

**Authors:** Hong-Mei Liu, Gong-Wei Zhang, Hong Yu, Xue-Fei Li, Hai Yu

**Affiliations:** 1 Department of Anesthesiology, West China Hospital, Sichuan University, Chengdu, 610041 China; 2 West China Fourth Hospital, Sichuan University, Chengdu, 610041 China; 3 Department of Anesthesiology, West China (Airport) Hospital, Sichuan University, Chengdu, 610072 China

**Keywords:** Mechanical power, Postoperative pulmonary complications, Thoracic surgery, One-lung ventilation

## Abstract

**Background:**

The role of mechanical power on pulmonary outcomes after thoracic surgery with one-lung ventilation was unclear. We investigated the association between mechanical power and postoperative pulmonary complications in patients undergoing thoracoscopic lung resection surgery.

**Methods:**

In this single-center, prospective observational study, 622 patients scheduled for thoracoscopic lung resection surgery were included. Volume control mode with lung protective ventilation strategies were implemented in all participants. The primary endpoint was a composite of postoperative pulmonary complications during hospital stay. Multivariable logistic regression models were used to evaluate the association between mechanical power and outcomes.

**Results:**

The incidence of pulmonary complications after surgery during hospital stay was 24.6% (150 of 609 patients). The multivariable analysis showed that there was no link between mechanical power and postoperative pulmonary complications.

**Conclusions:**

In patients undergoing thoracoscopic lung resection with standardized lung-protective ventilation, no association was found between mechanical power and postoperative pulmonary complications.

**Trial registration:**

Trial registration number: ChiCTR2200058528, date of registration: April 10, 2022.

**Supplementary Information:**

The online version contains supplementary material available at 10.1186/s12871-024-02562-1.

## Background

Postoperative pulmonary complications (PPCs) are common following lung resection surgery and remain a major determinant of postoperative morbidity and mortality [1]. Lung-protective ventilation strategies are strongly recommended during thoracic surgery to reduce the incidence of PPCs [2, 3]. Protective ventilation during one-lung ventilation (OLV) includes three parts: tidal volume (V_T_) 4–6 ml/kg of predicted body weight (PBW), positive end-expiratory pressure (PEEP) 5–10 cmH_2_O and the recruitment maneuver [4]. Nevertheless, the effect of isolated part on the pulmonary outcomes in the context of lung-protective ventilation remains controversial [5–8].

Recently, mechanical power (MP), that is the amount of energy per unit of time generated and converted to the respiratory system by the ventilator, combining volume, pressure, flow and respiratory rate (RR), has been proposed [9]. MP, as a single variable, has been hypothesized to help estimate the contribution of the different ventilator-related causes of lung injury and of their variations. Previous retrospective studies reported that exposure to higher MP was associated with increased risk of mortality in patients with acute respiratory distress syndrome (ARDS) [10–12]. Furthermore, MP normalized to lung size (MP normalized to PBW [norMP] or MP normalized to the compliance of respiratory system [MP/Crs]) has been suggested to have better discrimination power than the absolute MP value in ARDS patients [12, 13].

However, evidence regarding MP and postoperative pulmonary outcomes for surgical patients was rare and mixed with divergent conclusions [14–17]. A second analysis of a randomized clinical trial found that elevated MP during major noncardiothoracic surgery was independently correlated with increased risk of PPCs [15]. In contrast, a prospective observational trial (*n* = 30) suggested that there existed no link between MP and pulmonary complications after thoracic surgery [14].

Clinical trials exploring the effect of MP on PPCs after thoracic surgery are requested [18, 19]. Therefore, we designed a prospective, observational study to evaluate the association between MP during OLV and pulmonary complications after thoracoscopic lung resection surgery.

## Methods

### Study design

We performed a single-center, prospective observational study, which was approved by Ethical Committee of the West China Hospital of Sichuan University (Ethical Committee No.2021 (1580) and registered at the Chinese Clinical Trial Registry (ChiCTR2200058528, principal investigator: Hai Yu, date of registration: April 10, 2022. https://www.chictr.org.cn/showproj.html?proj=155531) before the first patient was enrolled. This report adhered to the applicable STrengthening the Reporting of OBservational studies in Epidemiology (STORBE) statement [20]. Written informed consent was obtained from all participants.

### Participants

Adult patients (aged 18 years or older) undergoing elective thoracoscopic lung resection surgery with a duration of OLV ≥ 1 h were included. The exclusion criteria were: American Society of Anesthesiologists classification 4 or above; previous history of neuromuscular diseases or brain trauma or brain injury; acute lung injury or ARDS or severe chronic obstructive pulmonary diseases or previous lung surgery; severe hepatic insufficiency or renal failure requiring dialysis.

### Perioperative management

Standard monitoring included electrocardiogram, non-invasive or invasive blood pressure, SpO_2_, end-tidal carbon dioxide (ETCO_2_), neuromuscular blockade and bispectral index (BIS) monitoring. Common or video double-lumen tube of the appropriate size (32–37 French) was chosen according to gender, height and chest radiograph. Correct placement of the common double-lumen tube was verified by fiberoptic bronchoscope both in the supine and lateral position. Intraoperative anesthetic management was at the discretion of the attending anesthesiologist according to routine practice. Anesthesia was maintained with volatile anesthetics or intravenous propofol targeting a BIS value between 40 and 60. The train-of-four (TOF) count was monitored every 15 min. Reversal of neuromuscular blockade was suggested with neostigmine of 0.04 mg/kg at a TOF count of at least 3. Multimodal analgesia including non-steroidal anti-inflammatory drugs, glucocorticoids, intercostal nerve blockade and patient-controlled intravenous analgesia was applied to maintain a numerical pain rating scale < 4 [21].

All participants were ventilated in volume control mode with a V_T_ of 7 ml/kg of PBW during two-lung ventilation (TLV) and 5 ml/kg during OLV. Other parameters applied during mechanical ventilation were: PEEP level was determined by the attending anesthesiologist according to clinical guidelines (5–10 cmH_2_O) [22]; inspiratory pause of 20% to obtain the end-inspiratory plateau pressure (Pplat); inspiration: expiration ratio of 1:2; RR adjusted to maintain ETCO_2_ between 35 and 45 mmHg; inspiratory oxygen fraction 1.0 during TLV and 0.4–0.5 during OLV to maintain SpO_2_ > 92%. Manual recruitment maneuvers were performed immediately after tracheal intubation, the restart of TLV and the end of surgery with a continuous positive airway pressure of 30 cmH_2_O (TLV) or 20 cmH_2_O (OLV) for 15 s.

If hypoxemia occurred (defined as SpO_2_ < 92% or PaO_2_ < 60 mmHg without evidence of incorrect tube placement, airway obstruction, or hemodynamic impairment), immediate rescue recruitment maneuvers were performed on the ventilated lung. If this maneuver failed to enhance oxygenation, we incrementally increased the FiO_2_ and applied continuous positive airway pressure of 5 cmH_2_O to the nonventilated lung.

### Outcome measures

The primary outcome was a composite of PPCs during hospital stay (respiratory infection, respiratory failure, pleural effusion, atelectasis, pneumothorax and bronchospasm) [23, 24]. Secondary outcomes included: the severity grade of pulmonary complications [25]; length of hospital stay; all-cause mortality during hospital stay and at postoperative day 30. The severity grade of PPCs was scored on a 0–5 scale, where 0 indicates no symptoms or signals of PPCs, grades 1–4 indicates successively worse forms of complications, and grade 5 indicates death before discharge (A description of the diagnosis of PPCs and the severity grade of PPCs is given in Appendix 1, Table S1 and Table S2).

Baseline characteristics, intraoperative surgery- and anesthesia-related data were recorded. Intraoperative mechanical ventilation parameters were collected at four timepoints: 1) T1-lateral position during TLV; 2) T2- lateral position after 10 min of OLV; 3) T3-lateral position after 1 h of OLV; 4) T4-TLV after surgery. Ventilation data was collected every hour during OLV. Outcomes were evaluated every afternoon by trained investigators during postoperative visits until discharge and at postoperative day 30 by telephone.

### Exposure

The primary exposure was MP measured after 1 h of OLV (T3). The following equation was applied to calculate MP, norMP and MP/Crs:

$$\mathrm{MP}\left(\mathrm J/\min\right)\;=\;0.098\;\times\;{\mathrm V}_{\mathrm T}\;\times\;\mathrm{RR}\;\times\;\left(\mathrm{Ppeak}-0.5\;\times\;\lbrack\mathrm{Pplat}-\mathrm{PEEP}\rbrack\right),\;\mathrm{where}\;\mathrm{Ppeak}\;\mathrm{was}\;\mathrm{the}\;\mathrm{peak}\;\mathrm{inspiratory}\;\mathrm{pressure};\;\mathrm{PBW}\;=\;50.0\;+\;0.91\;\times\left(\mathrm{height}\left[\mathrm{cm}\right]\right)-152.4)\;\mathrm{in}\;\mathrm{males},\;\mathrm{PBW}\;=\;45.5\;+0.92\;\times\left(\mathrm{height}\left[\mathrm{cm}\right]-152.4\right)\;\mathrm{in}\;\mathrm{females},\;\mathrm{norMP}\left(\times10^{-3}\;\mathrm J/\min/\mathrm{kg}\right)=\;\mathrm{MP}/\mathrm{PBW};\;\mathrm{Crs}\;=\;{\mathrm V}_{\mathrm T}/\left(\mathrm{Pplat}-\mathrm{PEEP}\right)$$

### Statistical analysis

Sample size was calculated using the rule of ten [26, 27]. Sample size = 10 × number of factors and cofactors/incidence of PPCs. On the basis of a 22% incidence of pulmonary complications after lung resection surgery [28, 29], 13 factors and a dropout rate of 5%, we estimated a sample size of 622 patients would be sufficient for this study.

Continuous variables were described as mean [standard deviation {SD}] or median [interquartile range {IQR}] and analyzed using the Student *t* test or the Mann–Whitney *U* test for normal or nonnormal distributions, respectively. Normality was tested using the Shapiro–Wilk test. Categorical variables were described as frequencies (percentages) and analyzed using Pearson’s test or Fisher’s exact tests. Data were analyzed with multivariable logistic regression, which provided odds ratios (OR) with 95% confidence intervals (CIs). Cofactors were included if they affected the outcome variable univariately (*P* < 0.1). If two factors were correlated (Variance inflation factor > 10), one of the factors would be excluded according to clinical considerations. Missing data were not addressed as < 5% of data for postoperative assessments were missing. A 2-tailed *P* value of < 0.05 was considered statistically significant. All analyses were conducted using IBM SPSS Statistics software (version 23.0).

## Results

**Fig. 1:**
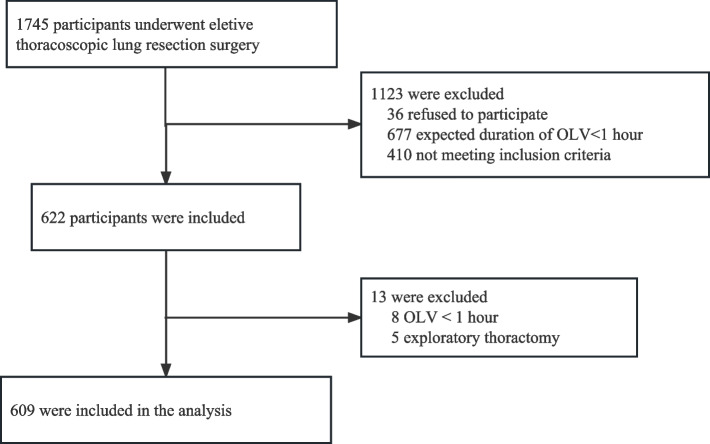
STORBE diagram. OLV: One-lung ventilation

**Fig. 2:**
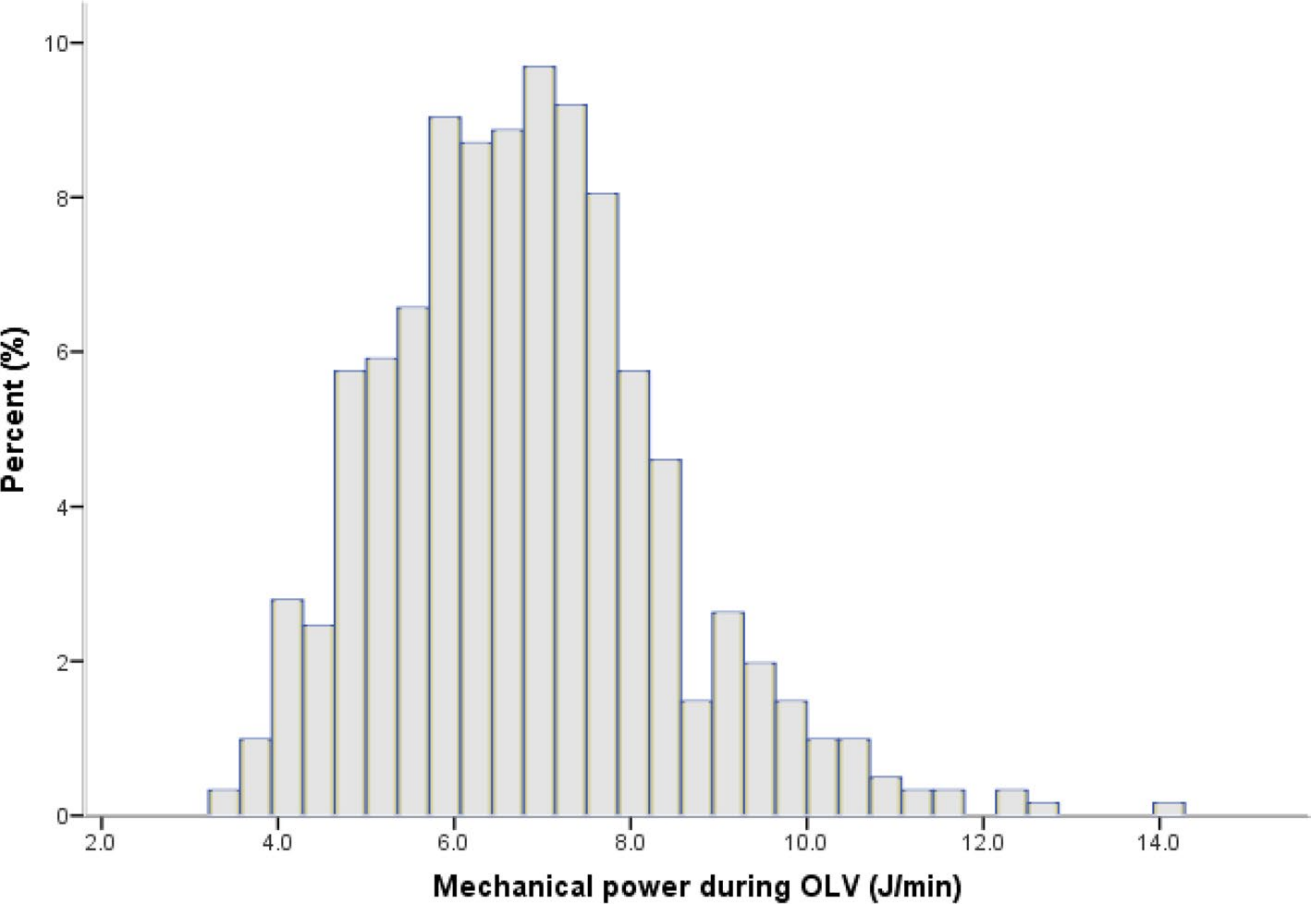
Histogram depicting distribution of mechanical power during one-lung ventilation

Between April 2022 and November 2022, we recruited 622 patients in total and 609 participants were analyzed in this study. Figure 1 illustrates details about the enrolled and excluded participants. Baseline characteristics of patients are shown in Table 1. In this study, median age was 55 years old and the majority of patients (68.1%) were female. Median duration of OLV was 90 min, thus ventilation parameters during OLV were only obtained twice in most patients. Ventilation characteristics of patients are presented in Table 2. The ventilation data at T4 of one patient were not collected. Ventilation parameters including MP at any study time-point were similar between patients with PPCs and patients without PPCs (Table 2). The distribution of MP during OLV is shown in Fig. 2.

**Table 1:** Baseline characteristics of 609 patients

Characteristics	All (*n* = 609)	PPCs(*n* = 150)	No PPCs(*n* = 459)	*P* value
Age; y	55(48–63)	57(49–67)	54(47–62)	**0.016**
Sex; female	415(68.1%)	89(59.3%)	326(71.0%)	**0.009**
BMI; kg/m^2^	23(21–25)	24(24–27)	23(21–24)	**< 0.001**
ASA physical status				**0.003**
1–2	548(90.0%)	125(83.3%)	423(92.2%)	
3	61(10.0%)	25(16.7%)	36(7.8%)	
Smoking status				0.499
Never	529(86.9%)	128(85.3%)	401(87.4%)	
Former	36(5.9%)	8(5.3%)	28(6.1%)	
Current	44(7.2%)	14(9.3%)	30(6.5%)	
ARISCAT risk score ≥ 26	439(72.1%)	117(78.0%)	322(70.2%)	0.074
Comorbidity				
COPD	23(3.8%)	7(4.6%)	16(3.5%)	0.470
Hypertension	115(18.9%)	36(24.0%)	79(17.2%)	0.072
Diabetes mellitus	35(5.7%)	10(6.7%)	25(5.4%)	0.550
Coronary artery disease	16(2.6%)	7(4.7%)	9(2.0%)	0.082
FEV1/FVC; %	80.4(77.2–84.5)	79.4(76.5–83.3)	80.8(77.4–85.1)	**0.012**
Surgical technique				0.700
Three-port VATS	232(38.1%)	55(36.7%)	177(38.6%)	
Uniportal VATS	377(61.9%)	95(63.3%)	282(61.4%)	
Type of surgery				0.369
Wedge resection	127(20.9%)	27(18.0%)	100(21.8%)	
Segmentectomy	242(39.7%)	57(38.0%)	185(40.3%)	
Lobectomy	240(39.4%)	66(44.0%)	174(37.9%)	
Duration of surgery; min	95(75–120)	109(84–129)	90(74–115)	**< 0.001**
Duration of OLV; min	90(70–115)	105(80–125)	85(70–110)	**< 0.001**
Total fluids; ml/kg/h	4.0(3.1–4.9)	4.0(3.1–4.9)	4.0(3.1–4.9)	0.942

Data are median [IQR] or n (%)

*BMI* Body mass index, *ASA* American Society of Anesthesiologists, *ARISCAT* Assess respiratory risk in surgical patients in Catalonia, *COPD* Chronic obstructive pulmonary disease, *FEV1* Forced expiratory volume in one second, *FVC* Forced vital capacity, *VATS* Video-assisted thoracoscopic surgery, *OLV* One-lung ventilation

**Table 2:** Characteristics of mechanical ventilation during surgery

	**All(*n* = 609)**	**PPCs(*n* = 150)**	**No PPCs(*n* = 459)**	***P* value**
V_T_/PBW				
T1	6.7(6.5–6.9)	6.7(6.5–6.9)	6.7(6.5–7.0)	0.438
T2	4.7(4.6–4.9)	4.7(4.6–4.9)	4.7(4.6–4.9)	0.635
T3	4.7(4.6–4.9)	4.7(4.6–4.9)	4.7(4.6–4.9)	0.591
T4	6.7(6.5–6.9)	6.7(6.5–6.9)	6.7(6.5–6.9)	0.410
RR				
T1	12(12–12)	12(12–12)	12(12–12)	0.478
T2	16(15–16)	16(15–16)	16(15–16)	0.027
T3	16(15–16)	16(15–16)	16(15–16)	0.089
T4	12(12–12)	12(12–12)	12(12–12)	0.375
Ppeak				
T1	18(16–21)	18(16–21)	18(16–21)	0.841
T2	23(20–25)	23(20–25)	23(20–25)	0.387
T3	23(21–25)	23(21–25)	23(21–25)	0.732
T4	20(17–22)	20(17–22)	20(17–22)	0.448
Pplat				
T1	15(13–17)	15(13–17)	15(13–17)	0.884
T2	18(16–20)	18(16–20)	18(16–20)	0.517
T3	18(16–20)	18(16–20)	18(16–20)	0.818
T4	16(14–18)	16(14–19)	16(14–18)	0.376
PEEP				
T1	5(5–10)	5(5–10)	5(5–10)	0.921
T2	5(5–10)	5(5–10)	5(5–10)	0.969
T3	5(5–10)	5(5–10)	5(5–10)	0.888
T4	5(5–10)	5(5–10)	5(5–10)	0.890
Driving pressure				
T1	8(7–9)	8(7–9)	8(7–9)	0.815
T2	11(9–12)	11(9–12)	10(9–12)	0.268
T3	11(9–13)	11(9–13)	11(9–12)	0.410
T4	9(8–11)	9(8–11)	9(8–11)	0.085
Crs				
vT1	46.2(38.6–54.5)	45.6(39.3–55.0)	46.5(38.4–54.4)	0.887
T2	23.5(19.9–29.3)	23.5(19.7–29.3)	23.5(20.0–29.3)	0.746
T3	23.4(19.3–28.7)	23.5(19.3–27.9)	23.3(19.4–29.0)	0.675
T4	39.0(32.5–46.9)	38.2(32.8–46.2)	39.4(32.3–47.1)	0.288
MP				
T1	6.05(4.87–7.11)	6.02(4.94–7.13)	6.06(4.85–7.10)	0.977
T2	6.39(5.38–7.53)	6.64(5.53–7.73)	6.31(5.30–7.52)	0.121
T3	6.75(5.73–7.69)	6.76(5.84–7.68)	6.72(5.68–7.69)	0.413
T4	6.43(5.25–7.51)	6.57(5.47–7.51)	6.36(5.14–7.52)	0.290
norMP				
T1	112.6(92.8–134.7)	109.7(92.6–137.3)	113.5(92.9–134.2)	0.808
T2	121.4(102.7–140.4)	122.9(107.1–141.8)	120.1(101.1–140.3)	0.241
T3	127.3(107.9–143.3)	129.0(109.6–143.2)	125.9(107.3–143.3)	0.427
T4	122.0(100.5–141.1)	121.1(101.3–140.4)	122.2(100.1–141.2)	0.650
MP/Crs				
T1	0.13(0.10–0.16)	0.13(0.10–0.16)	0.13(0.10–0.16)	0.869
T2	0.27(0.22–0.33)	0.28(0.22–0.34)	0.27(0.21–0.32)	0.131
T3	0.28(0.23–0.36)	0.29(0.23–0.36)	0.28(0.22–0.35)	0.276
T4	0.16(0.13–0.20)	0.17(0.14–0.21)	0.16(0.12–0.20)	0.131

Data are presented as median [IQR]

*V*_*T*_ Tidal volume, *PBW* Predicted body weight, *RR* Respiratory rate, *Ppeak* Peak inspiratory pressure, *Pplat* End-inspiratory plateau pressure, *PEEP* Positive end-expiratory pressure, *Crs* Compliance of the respiratory system, *MP* Mechanical power

**Fig. 3:**
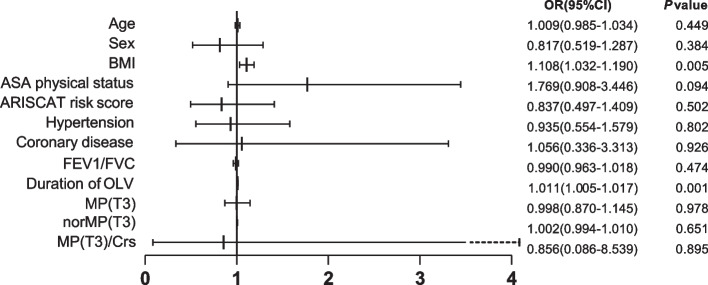
Odds ratio for postoperative pulmonary complications. ASA: American Society of Anesthesiologists; ARISCAT = Assess Respiratory Risk in Surgical Patients in Catalonia; FEV1/FVC: Forced expiratory volume in one second/Forced vital capacity; OLV: One-lung ventilation; MP: Mechanical power; Crs: Compliance of the respiratory system

The incidence of pulmonary complications after surgery during hospital stay was 24.6% (150 of 609 patients). The most common pulmonary complications were respiratory failure in 71 (11.7%) patients, atelectasis in 70 (11.5%) patients and respiratory infection in 18 (3.0%) patients. Results of the univariable analysis of the primary outcome are shown in Table 1. And there was no association between MP and in-hospital PPCs in the multivariable analysis (Fig. 3). In addition, MP was replaced by norMP and MP/Crs separately in multivariable analysis, and we did not detect association between norMP or MP/Crs and PPCs.

During hospital stay, 14 and 6 patients experienced mild and moderate PPCs, respectively. The association between MP and the severity grade of PPCs cannot be assessed due to insufficient data. The length of hospital stay in patients with PPCs was longer than patients without PPCs (5[4–7] vs 4[4, 5], *p* < 0.001). There were no deaths during hospital stay and at postoperative day 30.

## Discussion

In this study conducted in patients undergoing thoracoscopic lung resection surgery, there existed no link between MP and the incidence of PPCs during hospital stay in the context of lung-protective ventilation.

Ventilation characteristics keep changing over time during mechanical ventilation. According to the classical equation of MP proposed by Gattinoni et al. [9], the same MP can be achieved by different ventilation parameters, allowing MP a holistic indicator for monitoring mechanical ventilation. The effect of low tidal volume with or without PEEP on PPCs was uncertain, which indicated that MP was probably the determinant of pulmonary complications pathogenesis [5, 6].

Previous retrospective data suggested that elevated MP was associated with higher mortality in critically ill patients [11, 30–32]. Several clinical trials showed that the MP in ARDS patients receiving mechanical ventilation ranged from 15 to 29 J/min [11–13, 31]. Compared with ARDS patients, the absolute MP value was lower in surgical patients, though research about MP in surgical population was limited. Two clinical trials investigating general anesthesia outcomes reported that the MP in patients with PPCs or postoperative respiratory failure requiring reintubation was around 7.7 J/min [16, 17]. Karalapillai et al. [15] concluded that a higher MP was independently associated with higher risk of PPCs in surgical patients, and median MP of overall participants was 9.0 (7.0–11.4) J/min. However, so far, limited clinical data is available on the safe threshold of MP for developing PPCs in thoracic patients.

The incidence of PPCs in our study was 24.6%, which was on par with that observed in similar studies [23, 29]. Under lung-protective ventilation strategies including low tidal volume, optimal application of PEEP and recruitment maneuvers, we found MP was not associated with pulmonary complications after thoracoscopic lung resection surgery. However, Chiumello et al. [14] found that patients who developed PPCs had higher MP value during OLV (14.37 ± 8.19 vs 10.44 ± 2.82 J/min, *p* = 0.059) than patients without PPCs. Possible reasons for this discrepancy: 1) higher age (72 vs 55, y); 2) longer duration of OLV (137 vs 90, min); 3) patients with previous lung surgery (16.7% vs 0%); 4) larger V_T_ and RR during OLV. In addition, the median MP during OLV in patients with PPCs in our study was 6.76 J/min. Notably, this value was significantly lower than the corresponding MP value reported in Chiumello’s study (14.37 J/min) [14] and in Suleiman’s study (9.8 J/min) [33]. In this study, four patients exhibited a MP exceeding 12 J/min, the threshold previously suggested as detrimental in ARDS patients [34]. Among these, two patients subsequently developed PPCs. Though a higher intensity of OLV measured by MP was dose-dependently associated with postoperative respiratory failure, high absolute MP value may not result in adverse outcomes [33].

Based on these above and existed evidence about high MP in ARDS patients, we suspected that MP is a beneficial indicator for critically ill patients or patients requiring long-time surgery. In other words, MP can evaluate precisely patients’ prognosis when there existed great previous lung injuries or injuries caused by the ventilator.

To our knowledge, this is the largest prospective observational study to explore the association between MP and PPCs in thoracic surgical patients. Our findings could be generalized to ventilation approaches in thoracoscopic lung resection surgery using OLV. The present analysis also has some limitations. First, MP was determined by several ventilation variables, and the interaction among these variables during mechanical ventilation may confuse the association between MP and postoperative outcomes. In our study, the ventilator settings were similar among all participants, however, manipulating a single variable (such as PEEP), which correlates with variations in delivered MP, is likely a better approach to delineating its impact as Schujit et al. [35] investigated. Second, the result may not be wholly generalizable to other practices because the participants in our center were relatively young with healthy and underwent procedures of relatively short duration. Third, measurements of esophageal pressure were lacking. Therefore, MP calculating in our study was delivered by the ventilator to the whole respiratory system rather than lung separately. We used airway pressure instead of transpulmonary pressure to calculate MP, which was more accordant with clinical practice.

## Conclusions

In a population of patients undergoing thoracoscopic lung resection with standardized lung-protective ventilation, we did not detect an association between mechanical power and postoperative pulmonary complications.

### Supplementary Information

Supplementary Material 1.

## Data Availability

The datasets used and analyzed during the current study are available from the corresponding author on reasonable request.

## Abbreviations

ARDS: Acute respiratory distress syndrome
ARISCAT: Assess Respiratory Risk in Surgical Patients in Catalonia
ASA: American Society of Anesthesiologists
BIS: Bispectral index
CI: Confidence interval
ETCO_2_: End-tidal carbon dioxide
IQR: Interquartile range
MP: Mechanical power
OLV: One-lung ventilation
OR: Odds ratio
PBW: Predicted body weight
PEEP: Positive end-expiratory pressure
PPCs: Postoperative pulmonary complications
RR: Respiratory rate
SD: Standard deviation
STORBE: Strengthening the reporting of observational studies in epidemiology
TLV: Two-lung ventilation
TOF: Train of four
V_T_: Tidal volume
