# Supplementary material for: Association between mechanical power during one-lung ventilation and pulmonary complications after thoracoscopic lung resection surgery: a prospective observational study
Source: BMC Anesthesiol. 2024 May 17;24:176. doi: 10.1186/s12871-024-02562-1 (PMC11100229; doi:10.1186/s12871-024-02562-1)
Supplement: Supplementary file 1 — Supplementary Material 1. [file 12871_2024_2562_MOESM1_ESM.docx]

**Supplemental Table S1 Definitions of postoperative pulmonary complications**

| Complication | Definition |
| --- | --- |
| Respiratory infection | Patient has received antibiotics for a suspected respiratory infection and met one or more of the following criteria: new or changed sputum, new or changed lung opacities, fever, white blood cell count > 12×10^9^/L |
| Respiratory failure | Postoperative PaO_2_<60mmHg on room air, a ratio of PaO_2_ to inspired oxygen fraction <300mmHg or arterial oxyhemoglobin saturation measured with pulse oximetry < 90% and requiring oxygen therapy |
| Pleural effusion | Chest radiograph demonstrating blunting of the costophrenic angle, loss of sharp silhouette of the ipsilateral hemidiaphragm in upright position, evidence of displacement of adjacent anatomical structures or (in supine position) a hazy opacity in one hemithorax with preserved vascular shadows |
| Atelectasis | Lung opacification with a shift of the mediastinum, hilum or hemidiaphragm toward the affected area, and compensatory over-inflation in the adjacent non-atelectatic lung |
| Pneumothorax | Air in the pleural space with no vascular bed surrounding the visceral pleura |
| Bronchospasm | Newly detected expiratory wheezing treated with bronchodilators |

PaO_2_: partial pressure of oxygen in arterial blood.

**Supplemental Table S2 The severity grade of postoperative pulmonary complications**

| Grade | Criteria |
| --- | --- |
| Grade 0 | -No symptoms or signals |
| Grade 1 | -Cough, dry |
|  | -Microatelectasis: abnormal lung findings and temperature > 37.5°C without other documented cause; normal chest radiograph |
|  | -Dyspnea, not due to other documented cause |
| Grade 2* | -Cough, productive, not due to other documented cause  -Bronchospasm: new wheezing or preexistent wheezing resulting in a change in therapy  -Hypoxemia: SpO_2_ < 90% at room air  -Atelectasis: gross radiological confirmation (concordance of 2 independent experts) plus either temperature > 37.5°C or abnormal lung findings  -Hypercarbia (PaCO_2_ > 50mmHg), requiring treatment |
| Grade 3 | -Pleural effusion, resulting in thoracentesis  -Pneumonia: radiological evidence (concordance of 2 independent experts) plus clinical symptoms (two of the following: leucocytosis or leucopenia, abnormal temperature, purulent secretions), plus either a pathological organism (by Gram stain or culture) or a required change in antibiotics |
|  | -Pneumothorax  -Noninvasive ventilation, strictly applied to those with all of the following: a) SpO_2_ ≤ 92% under supplemental oxygen; b) need of supplemental oxygen > 5L min^-1^; and respiratory rate ≥ 30 bpm  -Reintubation postoperative or intubation, period of ventilator dependence does not exceed 48 hours |
| Grade 4 | -Ventilatory failure: postoperative ventilator dependence exceeding 48 hours, or reintubation with subsequent period of ventilator dependence exceeding 48 hours |
| Grade 5 | -Death |

* We only classified as grade 2 if two or more items in the grade 2 were present.

SpO_2_, pulse oxygen saturation; PaCO_2_, partial pressure of carbon dioxide in arterial blood.
